# Population-Level Exposure to Particulate Air Pollution during Active Travel: Planning for Low-Exposure, Health-Promoting Cities

**DOI:** 10.1289/EHP442

**Published:** 2016-10-07

**Authors:** Steve Hankey, Greg Lindsey, Julian D. Marshall

**Affiliations:** 1 School of Public and International Affairs, Virginia Tech, Blacksburg, Virginia, USA; 2 Humphrey School of Public Affairs, University of Minnesota, Minneapolis, Minnesota, USA; 3 Department of Civil and Environmental Engineering, University of Washington, Seattle, Washington, USA

## Abstract

**Background:**

Providing infrastructure and land uses to encourage active travel (i.e., bicycling and walking) are promising strategies for designing health-promoting cities. Population-level exposure to air pollution during active travel is understudied.

**Objectives:**

Our goals were *a*) to investigate population-level patterns in exposure during active travel, based on spatial estimates of bicycle traffic, pedestrian traffic, and particulate concentrations; and *b*) to assess how those exposure patterns are associated with the built environment.

**Methods:**

We employed facility–demand models (active travel) and land use regression models (particulate concentrations) to estimate block-level (*n* = 13,604) exposure during rush-hour (1600–1800 hours) in Minneapolis, Minnesota. We used the model-derived estimates to identify land use patterns and characteristics of the street network that are health promoting. We also assessed how exposure is correlated with indicators of health disparities (e.g., household income, proportion of nonwhite residents). Our work uses population-level rates of active travel (i.e., traffic flows) rather than the probability of walking or biking (i.e., “walkability” or “bikeability”) to assess exposure.

**Results:**

Active travel often occurs on high-traffic streets or near activity centers where particulate concentrations are highest (i.e., 20–42% of active travel occurs on blocks with high population-level exposure). Only 2–3% of blocks (3–8% of total active travel) are “sweet spots” (i.e., high active travel, low particulate concentrations); sweet spots are located *a*) near but slightly removed from the city-center or *b*) on off-street trails. We identified 1,721 blocks (~ 20% of local roads) where shifting active travel from high-traffic roads to adjacent low-traffic roads would reduce exposure by ~ 15%. Active travel is correlated with population density, land use mix, open space, and retail area; particulate concentrations were mostly unchanged with land use.

**Conclusions:**

Public health officials and urban planners may use our findings to promote healthy transportation choices. When designing health-promoting cities, benefits (physical activity) as well as hazards (air pollution) should be evaluated.

**Citation:**

Hankey S, Lindsey G, Marshall JD. 2017. Population-level exposure to particulate air pollution during active travel: planning for low-exposure, health-promoting cities. Environ Health Perspect 125:–534; http://dx.doi.org/10.1289/EHP442

## Introduction

Designing cities to promote active travel is a potential strategy to improve public health (HUD 2012; Nieuwenhuijsen and Khreis 2016). Two health-relevant factors associated with the built environment are physical activity and air quality (Abrams et al. 2012; Frank et al. 2006). Most prior research has isolated the built environment’s effects on these factors separately; a recent literature review emphasizes that assessing how the spatial patterns of these factors overlap and are distributed in urban areas is an important yet understudied question (Giles and Koehle 2014). Our research aims to help fill this gap.

Dense, walkable neighborhoods are associated with increased physical activity (Frank et al. 2005), partially owing to increased active travel [i.e., cycling and walking (Hankey et al. 2012; Oakes et al. 2007)]. Emerging research focuses on describing patterns of bicycle and pedestrian traffic on transportation networks—for example, designing traffic count programs (Hankey et al. 2014; Nordback et al. 2013) and building facility–demand models (Hankey and Lindsey 2016; Miranda-Moreno and Fernandes 2011; Schneider et al. 2012). Those findings highlight research and policy questions about how best to provide safe, health-promoting infrastructure for active travel (Loukaitou-Sideris et al. 2014; McDonald et al. 2014; Wilson et al. 2010).

Epidemiologic studies suggest that within-city spatial patterns of air pollution are important for health (Beelen et al. 2014; Crouse et al. 2015; Miller et al. 2007). Urban air quality is associated with the built environment (Bechle et al. 2011; Hankey et al. 2012; Stone et al. 2007), and transport micro-environments—especially while cycling or walking—are important exposure pathways (Dons et al. 2012; Int Panis et al. 2010). Exposure to traffic-related air pollution during active travel has been linked to a number of health indicators (Cole-Hunter et al. 2016; Kubesch et al. 2015; Strak et al. 2010; Weichenthal et al. 2011, 2014), and exploratory studies have assessed individual-level exposure on cycling routes (Cole-Hunter et al. 2012; Jarjour et al. 2013).

A few studies have explored spatial interactions between neighborhood “walkability” (i.e., characteristics in a neighborhood that may influence a resident’s likelihood of walking) and ambient air pollution concentrations (Frank and Engelke 2005; Hankey et al. 2012; Marshall et al. 2009); we are not aware of studies that compared spatial patterns of “bikeability” and ambient air pollution. The walkability-based studies found that few places in urban areas have both low levels of air pollution and high walkability (“sweet-spot” locations). Several health impact assessments have found that the individual-level health benefits (i.e., physical activity) outweigh risks (i.e., air pollution, accidents) for hypothetical shifts to active travel (de Hartog et al. 2010; Doorley et al. 2015; Macmillan et al. 2014; Mueller et al. 2015; Rojas-Rueda et al. 2011). However, an understudied topic is the relative exposure of population-level flows of cyclists and pedestrians to air pollution [i.e., exposure where people actually walk and bike (traffic flows) rather than the characteristics of neighborhoods that influence their likelihood to walk (“walkability”) or bike (“bikeability”)]. [Several prior studies explore walkability and bikeability (Cole-Hunter et al. 2015; Frank et al. 2006; Winters et al. 2013).]

In this paper we used model-derived spatial estimates of bicycle and pedestrian traffic volumes and particulate air pollution concentrations in Minneapolis, Minnesota, to assess population-level patterns of exposure during active travel. We identified areas that are overall health-promoting and explored aspects of how best to design low-exposure transportation networks and neighborhoods (e.g., moving bicycle facilities away from high-traffic roads). Our analysis is one of the first to assess urban-scale exposure based on spatial estimates of actual rates of active travel (rather than “walkability”/“bikeability”). Our findings inform efforts to promote healthy transportation planning decisions, especially for approaches that encourage active travel on high-traffic roads (see the discussion of “Complete Streets,” below).

## Methods

Our analysis is based on previously published modeling approaches for Minneapolis: *a*) facility–demand models of bicycle and pedestrian traffic volumes (Hankey and Lindsey 2016) and *b*) land use regression (LUR) models of on-road particulate air pollution concentrations (Hankey and Marshall 2015). We used these models to generate estimates of active travel and particulate air pollution for every block in Minneapolis (*n* = 13,604) during the afternoon rush hour (1600–1800 hours). (Particulate measurements and traffic counts used to develop these models were collected during the afternoon and in autumn; thus, our results should be interpreted for this time period.) We assessed how exposure varies by characteristics of the road network and features of the built environment. Our approach offers unique insight into designing health-promoting cities in two ways: *a*) Our model-derived estimates of active travel describe where people actually walk or bike (rather than “walkability” or “bikeability”), and *b*) our LUR models are able to discern small-scale changes in particulate concentrations (i.e., spatial resolution: ~ 100 m) that a sparsely distributed regulatory monitor network cannot. By comparing the spatial patterns of active travel and particulate concentrations we were able to assess patterns of population-level exposure (i.e., for all walkers and bikers).

### Spatial Models of Bicycle and Pedestrian Traffic

Our spatial estimates of bicycle and pedestrian traffic are derived from facility–demand models that estimate traffic volumes based on land use, demographic, and weather-related variables (Hankey and Lindsey 2016) (Table 1). The models use afternoon rush-hour (1600–1800 hours) volunteer-based counts (954 observations; 471 locations; years 2007–2014) of cyclists and pedestrians (collected by the City of Minneapolis) as the dependent variable; few traffic counts were available for other time periods. We assembled independent variables (e.g., land use, demographic) at each count location based on network buffers of varying spatial scale; conventional stepwise linear regression was used for model-building. Model adjusted *R* ^2^ was 0.46 (0.50) for the bicycle (pedestrian) models. For further details on model development see Hankey and Lindsey (2016).

**Table 1:** Spatial models used here to estimate particulate concentrations and rates of active travel.

Study	Dependent variable	Observations	Model output	Model adj *R*^2^
Hankey and Lindsey 2016^*a*^	Bicycle counts Pedestrian counts	957 (471 locations)	Bicycle and pedestrian traffic volumes	Bike: 0.46 Ped: 0.50
Hankey and Marshall 2015^*a*^	Concentrations of particulate matter	1,101 locations along mobile routes	Concentrations of particle number (PN), black carbon (BC), and PM_2.5_	PN: 0.48 BC: 0.42 PM_2.5_: 0.49
^***a***^Models are based on data collected in the autumn and during afternoon rush-hour 1600–1800 hours.				

### Spatial Models of Air Quality

We applied previously published LUR models to estimate afternoon rush-hour (1600–1800 hours) particulate concentrations for all streets and trails in Minneapolis (Hankey and Marshall 2015) (Table 1). We measured and modeled three aspects of particulate air pollution: *a*) particle number (PN) concentration, a representation of ultrafine particles; *b*) black carbon (BC) mass concentration; and *c*) fine particulate matter (≤ 2.5 μm; PM_2.5_) mass concentration. The LUR model building employed two main inputs: *a*) mobile monitoring (i.e., measurements made while cycling along prescribed routes) during afternoon (1600–1800 hours) rush hour and *b*) land use variables assembled at varying spatial scales. The LUR models predict concentrations for surface streets and for off-street trails. We do not make predictions for on-freeway concentrations (walking and biking are typically illegal on highways); however, all street types (including length of freeway miles within a buffer) were included as potential variables during model-building). Adjusted *R* ^2^ values for the models were 0.42–0.49 among the pollutants. To match the estimates of air pollution and active travel, we estimated concentrations at the midpoint of each street segment (mean block length in Minneapolis, ~ 120 m) resulting in 13,604 point estimates of particulate concentrations, bicycle traffic, and pedestrian traffic for use in our spatial comparison.

### Spatial Analyses of Exposure During Active Travel

We performed three analyses to inform planning decisions on the built environment, infrastructure, and health promotion: *a*) identify “sweet spot” (i.e., high active travel and low particulate concentration) city blocks; *b*) explore trends in population-level exposure by characteristics of the road network (e.g., street functional class; proximity to major roads) to inform design of low-exposure bicycle and pedestrian networks; and *c*) assess whether features of the built environment (e.g., population density, land use mix, open space, retail area) and indicators of health disparities (e.g., household income, proportion of nonwhites) are correlated with exposure during active travel.

***Identifying “sweet spot” neighborhoods.*** A core motivation for combining outputs from our models was to explore patterns of population-level rather than individual-level exposure during active travel. For example, if one is only interested in individual-level exposure (e.g., when choosing a cycling route or estimating exposure for survey participants), then a concentration surface is sufficient information. However, a key question is how exposure patterns change at the population level. For example, when considering population health (or disparities in health), an area with moderate concentrations and high levels of active travel could be more important than an area with high concentrations but few people. To explore this issue we mapped four types of city blocks based on highest/lowest quartiles for model-generated estimates of particulate concentrations and rates of active travel: *a*) “sweet spot” (high active travel, low particulate concentration); *b*) “sour spot” (low active travel, high particulate concentration); *c*) “active and exposed” (high active travel, high particulate concentration); and *d*) “inactive and clean” (low active travel, low particulate concentration). We compared commonalities within neighborhood types that may inform design of “sweet spot” neighborhoods.

***Designing low-exposure bicycle and pedestrian networks.*** We explored trends in exposure during active travel that may be important for designing low-exposure bicycle and pedestrian networks. We stratified our model output by street functional class and proximity to major roads, with the goal of identifying where small shifts in bicycle and pedestrian infrastructure (e.g., locating cycling infrastructure away from high-traffic roads) may yield reductions in exposure. Identifying the exposure impact of these shifts may help policy makers choose between strategies that call for encouraging active travel on high-traffic roads (e.g., see “Complete Streets” discussion below) versus on residential streets (e.g., bicycle boulevards) or off-street trails. We identified and mapped blocks that met the following criteria: *a*) were on local roads within 200 m (i.e., the 75th percentile of block length in Minneapolis; mean block length is ~ 120 m) of a high-traffic road (i.e., a road classified as arterial or collector), and *b*) relative to the nearest high-traffic road, had at least a 15% reduction in PN or BC concentrations.

***Health-promoting features of the built environment.*** We stratified our model estimates by two types of built environment variables to assess patterns of exposure: *a*) variables commonly used in “walkability” indices (e.g., population density, land use mix) and *b*) land use variables that were correlated with active travel in our facility–demand models (e.g., open space, retail, and industrial area). See Table S1 for a summary of the land use variables.

An important aspect of planning for health-promoting cities is to ensure that healthy neighborhoods are distributed equitably among the population. Following previous studies (Brooks and Sethi 1997; Clark et al. 2014; Marshall 2008; Miranda et al. 2011; Schweitzer and Zhou 2010), we stratified our model results by household income and race to inform whether health disparities exist for our population (i.e., pedestrians and cyclists).

## Results

To summarize our findings, we first describe the raw model output: spatial patterns in active travel and air pollution concentrations; then we explore how combining model outputs can inform planning of health-promoting cities. Descriptive statistics of the model output are in Table S2.

### Spatial Patterns of Bicycling and Walking

**Figure 1:**
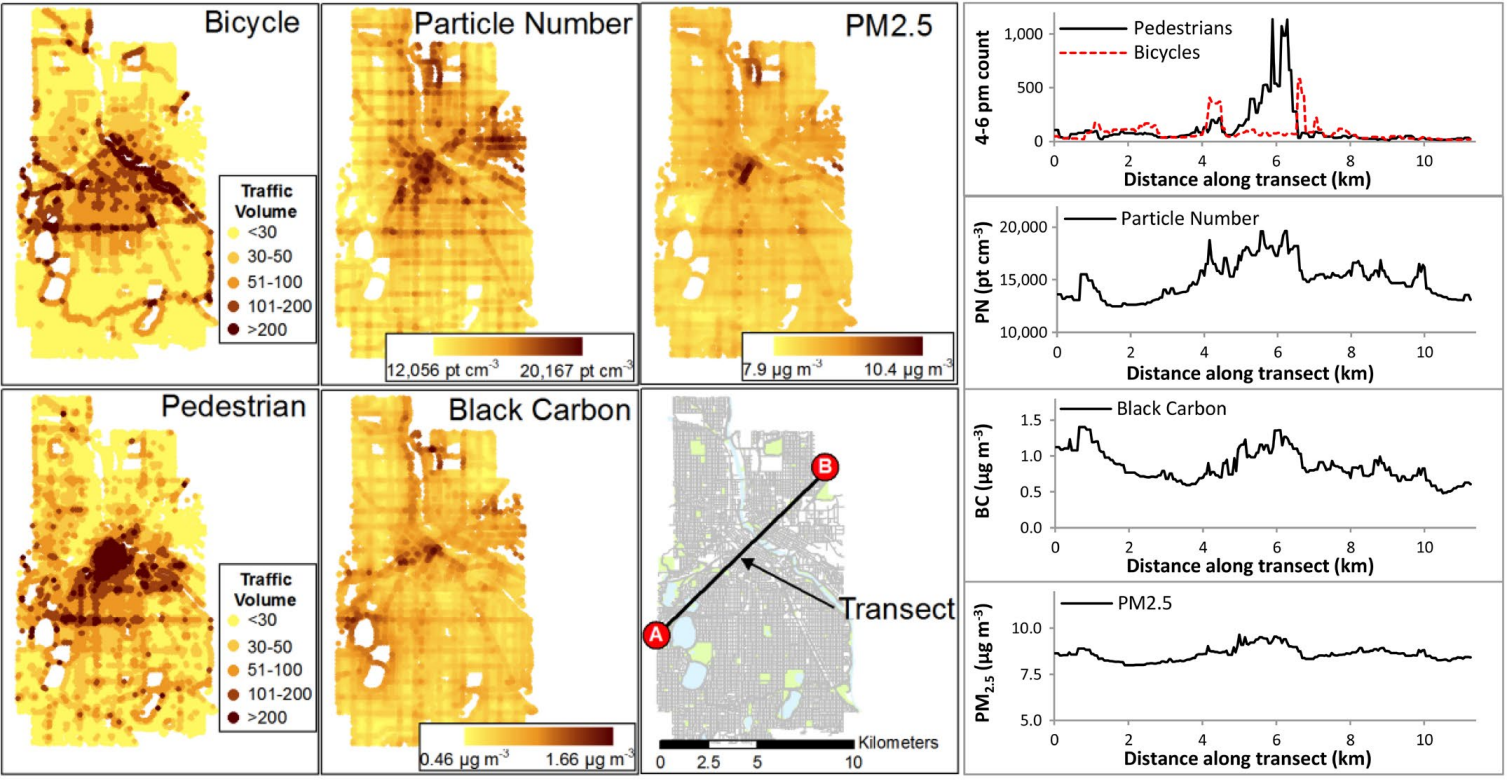
Spatial estimates of active travel and particulate air pollution for afternoon (1600–1800 hours) rush-hour in Minneapolis, MN. Abbreviations: BC, black carbon; PN, particle number; pt, particle. A sample transect shows spatial variability of each pollutant and mode. The transect plots (right-panel) correspond to tracing the transect from point A (left) to point B (right). Underlying street and land use data are from Minnesota Geospatial Commons (https://gisdata.mn.gov/).

We generated estimates of rush-hour (1600–1800 hours) bicycle and pedestrian traffic for all streets and trails in Minneapolis (Figure 1). As expected, given the significance of variables in the models, the maps reflect the importance of street functional class (higher traffic on arterials and collectors) and features of the built environment (e.g., higher traffic near retail areas and open space). These maps also highlight differences between bicycle and pedestrian traffic: *a*) Off-street trails generally have larger relative volumes for cyclists than for pedestrians, and *b*) pedestrian traffic is more tightly clustered around retail corridors and activity centers [e.g., near the Central Business District (CBD) and along major transportation corridors] than is bicycle traffic. In general, rates of active travel were greatest near the CBD and decreased as a function of distance from the CBD. Figure 1 also shows spatial variability in bicycle and pedestrian traffic for a sample transect across the city. The transect plot highlights that the largest differences in traffic are between locations near the CBD and outer-lying locations; local variability among street-types (i.e., major vs. local streets) and facility types (e.g., streets with no bike facility vs. streets with a bike lane or off-street trail) are also noticeable.

### Spatial Patterns of Particulate Air Pollution

Model-generated estimates of afternoon rush-hour particulate concentrations are shown in Figure 1 (concentrations along the same transect used for bicycle and pedestrian traffic are also shown for each pollutant). Concentrations are more spatially variable for PN and BC than for PM_2.5_. In general, concentrations are elevated in areas with dense networks of major roads in conjunction with activity centers (e.g., freeways near the CBD or arterials serving retail corridors), near significant emission sources (industrial and railway areas), and lower in areas that are farther from traffic sources (open space and on low-traffic roads). The magnitude of spatial variability differs among pollutants, but otherwise the spatial patterns are broadly similar among pollutants. White spaces in Figure 1 are locations with no or few roads (e.g., lakes, rivers, parks, railways).

### Spatial Analyses of Exposure During Active Travel

We next present our three spatial analyses based on stratifying model output by attributes of neighborhood built environment, infrastructure, and demographics. We then discuss how analyses that account for both health determinants simultaneously could be used to plan for health-promoting cities.

**Figure 2:**
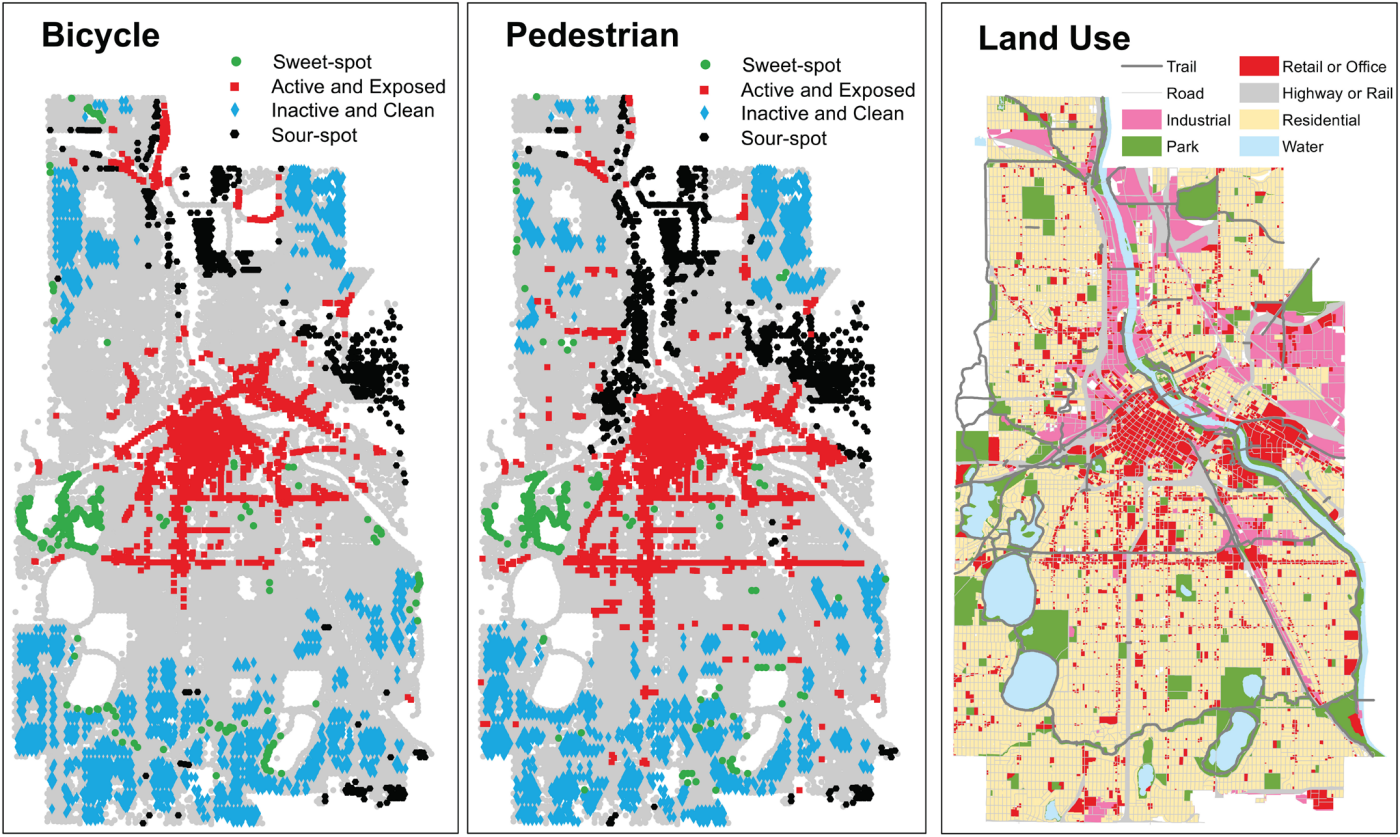
Block type as defined in Table 2 for bicycle (left panel) and pedestrian (middle panel) traffic (right panel: land use pattern). Block types were classified if inclusion criteria were met for two of three pollutants; see Figures S1–S3 for individual pollutants. Underlying street and land use data are from Minnesota Geospatial Commons (https://gisdata.mn.gov/).

***Identifying “sweet spot” neighborhoods.*** A small proportion of blocks are “sweet spot” (2–3%) or “sour spot” (3–7%); more blocks were classified as “active and exposed” (9–11%) or “inactive and clean” areas (9–12%). [If pollution and physical activity were spatially uncorrelated, then each of the four groups in the previous sentence would occupy 6% (i.e., 1/16) of the blocks.] An important finding is that a significant proportion of active travel occurs on “active and exposed” blocks (20–44%); more active travel occurs on sweet spot (3–9%) blocks than on sour spot (1–2%) blocks. “Sweet spot” blocks were located *a*) near, but just outside, the CBD or *b*) on off-street trails near lakes or parkways. These findings are consistent with previous studies of walkability and air pollution in Vancouver, BC, Canada (Marshall et al. 2009) and Los Angeles, California (Hankey et al. 2012). Table 2 gives a summary of each neighborhood category; Figure 2 maps each category for locations that met inclusion criteria for two of the three pollutants (see Figures S1–S3 for maps of individual pollutants).

**Table 2:** Blocks defined by spatial patterns of active travel and particulate concentrations.

Block type	Active travel	Air pollution	Mode of travel	Percentage of blocks^*a*^			Percentage of active travel^*a*^		
				PN	BC	PM_2.5_	PN	BC	PM_2.5_
Sweet spot: high active travel and low exposure to air pollution.	Highest quartile	Lowest quartile	Bicycles	3	2	3	7	5	9
			Pedestrians	2	3	3	3	4	4
Active and exposed: high active travel exposures.	Highest quartile	Highest quartile	Bicycles	10	10	9	21	27	20
			Pedestrians	11	11	10	44	38	41
Inactive and clean: blocks could benefit from increased active travel while keeping pollution low.	Lowest quartile	Lowest quartile	Bicycles	12	10	11	4	3	3
			Pedestrians	11	9	10	3	3	3
Sour spot: blocks with lowest health benefit for active travel and pollution.	Lowest quartile	Highest quartile	Bicycles	3	4	4	1	1	1
			Pedestrians	6	5	7	2	1	2
All other areas: blocks not classified in one of the four categories above.	—	—	Bicycles	72	74	73	67	64	67
			Pedestrians	70	73	70	48	54	50
^***a***^In each cell for percentage of blocks and percentage of active travel, values for bicycles are the top value, and pedestrians the bottom value.									

Most blocks (~ 72%) and most of the active travel [~ 50% (walking), ~ 65% (biking)] did not fall into any of the four categories defined in Table 2. [If travel and pollution were spatially uncorrelated, 75% of blocks or of travel (i.e., 12/16) would not fall into any of the four categories.] Analyses above highlight areas that do especially well (or poorly) for each factor (i.e., active travel or air pollution); however, blocks that were unclassified (i.e., are not in one of the four categories) may be important because they include places where moderate shifts in one factor may push those neighborhoods into one of the highlighted groups (e.g., if levels of active travel are increased on a road that has low particulate concentrations but moderate rates of active travel, then that road would shift to the “sweet spot” category). Many of the unclassified blocks are in residential neighborhoods; as such, these places may require different strategies to shift patterns of exposure. For example, active travel in residential areas might be predominantly recreational, whereas active travel in the CBD (where exposure is high) might be predominantly utilitarian. This difference may be important for encouraging activities that are overall health-promoting.

**Figure 3:**
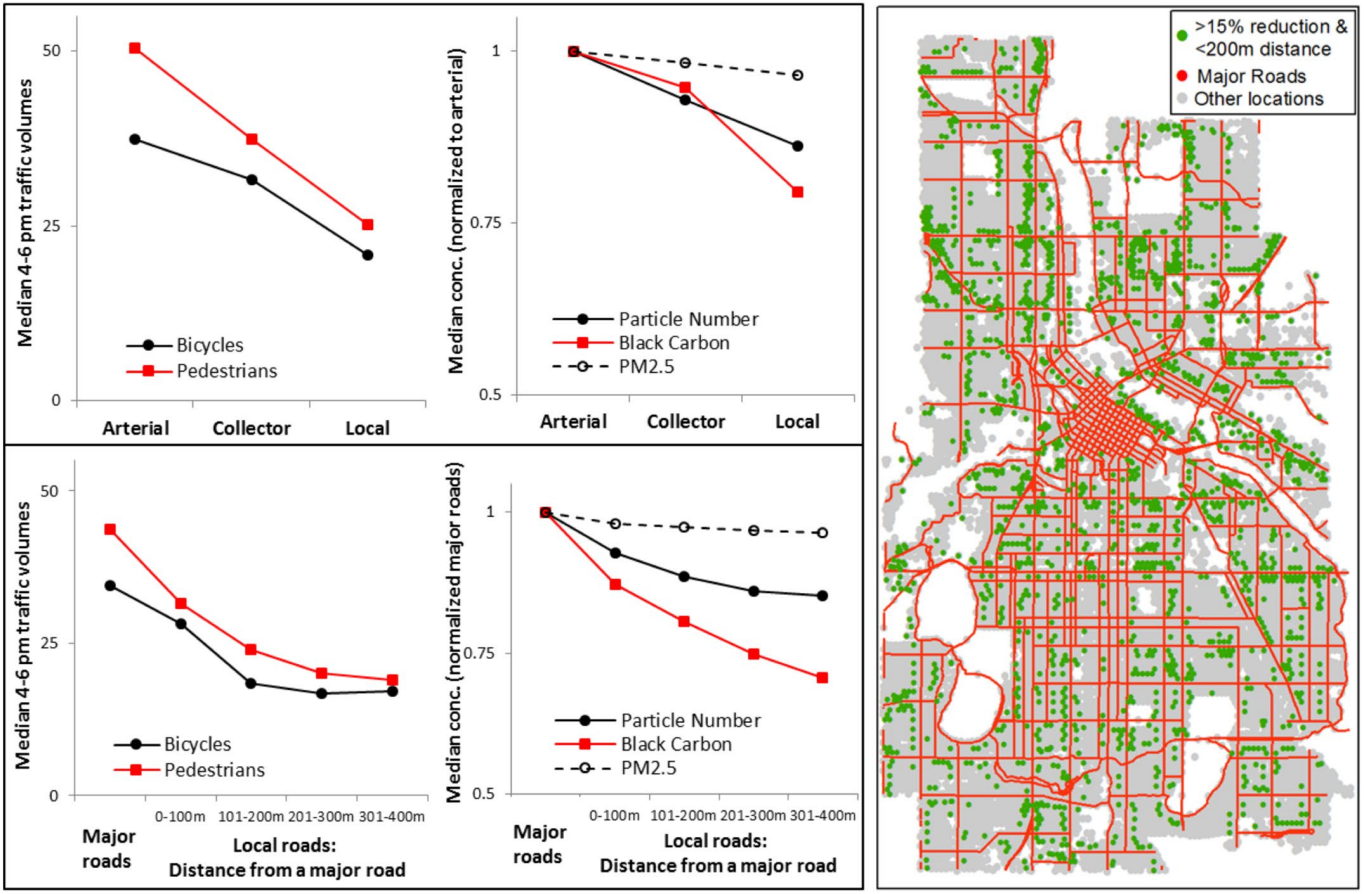
Median bicycle and pedestrian traffic volumes and particulate concentrations (conc.) by street functional class (upper-left panel) and distance from a major road (bottom-left panel). Right panel: Locations that are within 200 m of a major road (75th percentile distance for city blocks in Minneapolis) and, relative to that road, had at least a 15% reduction in PN or BC concentration. Underlying street and land use data are from Minnesota Geospatial Commons (https://gisdata.mn.gov/).

***Designing low-exposure bicycle and pedestrian networks.*** Particulate concentrations and active travel are correlated with street functional class (Figure 3). Median bicycle (pedestrian) traffic volumes were highest on arterials [19/hr (25/hr)] and decrease with street functional class [collectors: 16/hr (19/hr); local: 11/hr (13/hr)]. Bicycle volumes are highest on off-street trails (44/hr; 22/hr for pedestrians; Figure S4). Median particulate concentrations correlate with motor vehicle traffic. Concentration differences between low- and high-traffic roads averaged ~ 17% for PN and BC, ~ 3% for PM_2.5_.

In our data, a substantial proportion of cycling (29%) and walking (49%) occurs on high-traffic roads that are also the most polluted. However, it may be possible to target shifts in the bicycle and pedestrian network to slightly shift spatial patterns of active travel and reduce exposure to air pollution. For example, Minneapolis is currently building a network of bicycle boulevards (i.e., local roads with traffic-calming measures (e.g., speed bumps, bump outs, one-way vehicle traffic) that discourage vehicle traffic (but do not prohibit it) while encouraging bicycle traffic. One question that arises is what impact shifting active travel from major (high-traffic) roads to adjacent local roads may have on exposure. To shed light on that question, we summarized bicycle and pedestrian traffic, and particulate concentrations by *a*) major roads and *b*) local roads at specific distances from the nearest major road (Figure 3).

Active travel and particulate concentrations are highest on major roads and decrease steadily as the distance from a major road increases. For the bicycle boulevard example described above, shifting traffic over one block corresponds to an estimated average decrease in afternoon rush-hour exposure concentrations of 11% for PN, 19% for BC, and 3% for PM_2.5_. Figure 3 also includes a map of blocks that could be candidates for shifting active travel away from high-traffic roads by our criteria: local roads that are one block (i.e., within 200 m) from the nearest major road and have a 15% reduction in PN or BC concentration relative to that major road. This procedure identified 1,721 blocks (~ 20% of all local roads) that would be potential candidates for shifting active travel away from high-traffic roads that would yield ≥ 15% reductions in air pollution exposure. Incorporating this information in decisions about how to locate bicycle and pedestrian infrastructure may help planners work towards the goal of low-exposure networks, especially for policies such as “Complete Streets” which may not have flexibility to shift funding for bicycle and pedestrian infrastructure to adjacent corridors.

***Health-promoting features of the built environment.*** We stratified our model estimates by two factors commonly cited in “walkability” or “bikeability” metrics (population density; land use mix), three land use factors that were significant in our facility–demand models (retail, open space, and industrial area), and two indicators of health disparities (household income, percent of nonwhite residents).

**Figure 4:**
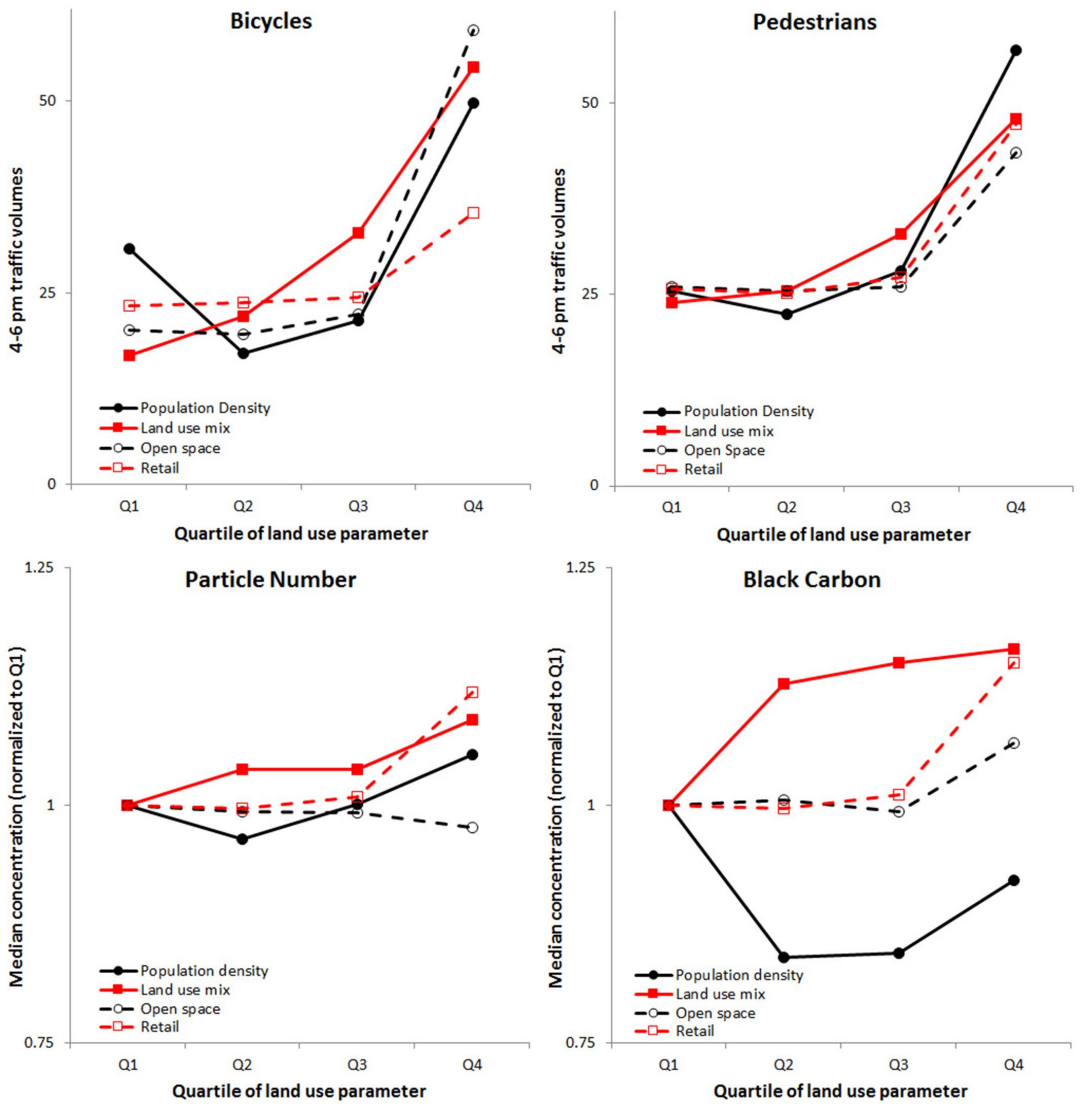
Median bicycle and pedestrian traffic volumes and particulate concentrations (PN and BC) by quartile (Q) of population density, land use mix, open space area, and retail area. See Figures S5–S10 for other land uses and pollutants.

Land use mix, population density, open space area, and retail area were associated with increased active travel (Figure 4). Bicycle and pedestrian traffic volumes were about two times higher in areas that were in the highest (Q4) vs. lowest (Q1) quartile of each variable suggesting that dense neighborhoods with mixed land use, open space, and retail area are correlated with higher rates of active travel. Particulate concentrations increased with increasing land use mix [9% (16%) increase for PN (BC) from Q1 to Q4] and retail area [12% (15%) increase for PN (BC)]; trends were mixed for population density [5% increase (PN), 8% decrease (BC)] and open space area [2% decrease (PN), 7% increase (BC)]. (See Figures S5–S8 for PM_2.5_ results; absolute concentrations are in Figure S9.) Although rates of active travel generally increased with increased population density, the lowest quartile had slightly elevated levels of active travel. This result could potentially be the result of certain high-use areas being located in low density areas, for example, in downtown Minneapolis (low number of residents). Industrial area had overall low rates of active travel yet was associated with increased particulate concentrations (see Figure S10).

Equitable distribution of access to health-promoting places is an important health-promoting policy goal (Clark et al. 2014; Miranda et al. 2011; Schweitzer and Zhou 2010). To explore this issue, we stratified our model estimates by median household income and proportion of nonwhite residents (see Figures S11–S12). Active travel and particulate concentrations were moderately higher for low-income blocks; neither factor varied much by proportion of nonwhite residents. The trends by household income may suggest that a disproportionate share of the air pollution exposure burden during active travel is occurring in low-income neighborhoods. Available demographic variables describe the residents of a neighborhood, but not necessarily the population traveling in that location.

## Discussion

Our work sheds light on population-level spatial patterns of exposure to air pollution during active travel that may be important for planning low-exposure cities that are overall health protective. Our analyses incorporate output from existing models of active travel and particulate air pollution. A key advantage to our approach is that we explored exposure patterns based on modeled estimates of traffic volumes where people actually walk and bike (traffic flows) rather than only physical indicators of walkability or bikeability. Our spatial models are for specific pollutants, times of day, and months of the year; future research could build on our approach by developing models with more spatial and temporal precision. Additional extensions of our research could replicate our results in other study locations or evaluate specific interventions or policy prescriptions.

We developed models of bicycle and pedestrian traffic only for the afternoon rush-hour (1600–1800 hours) because traffic counts are unavailable for other time periods. As a sensitivity analysis we explored an alternate scenario using a morning rush-hour (0700–0900 hours) particulate concentration surface. Spatial variability of air quality is greater in magnitude in the morning. Using the morning rush-hour particulate surface mostly exacerbated the patterns in exposure compared to the base-case (i.e., afternoon) analysis. More details on the sensitivity analysis are in Figures S13–S15; a useful future research question is to explore how spatial patterns of exposure change by time of day.

Our findings indicate that areas with high levels of population density, land use mix, open space area, and retail area have higher rates of active travel, which is consistent with previous studies (Frank et al. 2005; Miranda-Moreno and Fernandes 2011; Oakes et al. 2007). However, trends in particulate concentrations were less clear by type of land use. Particulate concentrations seemed to increase (or remain unchanged) with increases in each land use factor; PN (BC) concentrations decreased slightly as open space (population density) increased. For particulate concentrations, it is likely that the spatial location of the land use may play a larger role than the land use itself; for example, being located near a freeway, downtown, or in a residential neighborhood may be a more significant factor in determining the local exposure concentration than the immediate land use at that specific location. Therefore, a planning goal may be to increase pocket areas (i.e., on the block or multi-block scale) of high land use mix, open space, or retail uses in residential areas to increase rates of active travel while mitigating exposure to air pollution. This idea may be especially of interest for areas such as the “inactive and clean” blocks defined above. A limitation of our approach is that we analyzed cross-sectional data sets and stratified by land use variables that sometimes were selected in our spatial models. We therefore were not able to infer causality from our results. However, despite this limitation, our work sheds light on how urban planners might allocate future development (i.e., land use patterns) and bicycle and pedestrian infrastructure (i.e., low-exposure networks) with the goal of designing health-promoting cities.

We found that shifting active travel away from major roads to adjacent local roads could yield exposure reductions; this finding is consistent with previous studies (Cole-Hunter et al. 2012; Jarjour et al. 2013). Shifting active travel to adjacent streets (e.g., by strategically locating cycling infrastructure) may be a more realistic goal for cyclists than for pedestrians because pedestrians travel shorter distances and are less likely to follow alternative routes to destinations. In situations where exposure to air pollution is higher than acceptable for pedestrians, strategies that remove the pollution from the pedestrians may be of interest—for example, reducing bus traffic or the number of stops along a corridor with high pedestrian traffic (while relocating those stops and routes in close proximity to the pedestrian corridor). The finding that bicycle traffic was highest on off-street trails suggests that cyclists may adjust routes to use enhanced infrastructure.

Exposure to air pollution during transport is only one component of overall exposure. A recent study of 62 participants in Belgium found that ~ 6% of participant’s time was spent in transport and accounted for ~ 30% of total inhaled dose of BC (Dons et al. 2012). To put our findings in the context of overall exposure, we used a “back of the envelope” calculation to estimate the overall exposure reduction for a hypothetical cyclist who cycles frequently and chooses to move one block away from major roads to low-traffic roads while cycling. We considered the following scenario: *a*) Moving over one block represents a 15% reduction in BC (Figure 3), *b*) two-thirds of all trips for this individual are by bicycle, and *c*) for each trip it is possible to move to an adjacent, low-traffic road for 90% of the route (i.e., the origin and destination remain on high-traffic roads). Given these assumptions, we estimated the overall exposure reduction from this shift in cycling route as: 30% (total BC dose in transport) × 15% (exposure reduction from moving to low-traffic roads) × 66% (share of trips by bicycle) × 90% (share of route that it is feasible to move to a low-traffic road) = ~ 3% reduction in total exposure (~ 9% reduction in exposure during transport).

The overall reduction in exposure for this example is modest. However, if planners can encourage this type of shift for a large segment of the population (e.g., by considering exposure when locating infrastructure and future development patterns), the aggregate effects (i.e., reductions in population-level dose) could be noteworthy. Additionally, reducing exposure to one hazard (i.e., air pollution) could also include other health beneficial outcomes—for example, lower probability of an accident (i.e., low-traffic roads offer fewer interactions with motor vehicles) and increased physical activity (i.e., by cycling a slightly farther difference)—without the costs of implementing emission reductions.

## Conclusions

We employed previously published spatial models to estimate block-level rates of active travel and particulate concentrations to assess population-level exposure during active travel in Minneapolis, Minnesota. Only ~ 2–3% of blocks were “sweet spot” blocks (high active travel, low air pollution); these blocks were mostly located *a*) near but just outside of the CBD or *b*) on off-street trails. Active travel and particulate concentrations correlated with street functional class and proximity to high-traffic roads (leading to a spatial mismatch: 20–44% of active travel occurred on the “active and exposed” blocks). Our findings suggest that minor shifts to the bicycle and pedestrian network may reduce overall exposure, for example, by moving cyclists away from pollution by strategic location of bicycle infrastructure on low-traffic roads and/or moving pollution away from pedestrians by shifting the location of emission sources (e.g., bus routes or stops). We found that the highest rates of active travel are in neighborhoods with high levels of population density, land use mix, open space, and retail area. However, trends in particulate concentrations varied for those land use factors; particulate concentrations increased with land use mix and retail area and were mostly unchanged with population density and open space area. Our results suggest that not only does the type of land use matter, but so too does the spatial location of the land use. This research may aid in planning for low-exposure infrastructure and development for cyclists and pedestrians to support health-promoting transportation choices.

## Supplemental Material

(2.5 MB) PDF
