# Supplementary material for: Population-Level Exposure to Particulate Air Pollution during Active Travel: Planning for Low-Exposure, Health-Promoting Cities
Source: Environ Health Perspect. 2016 Oct 7;125(4):527–34. doi: 10.1289/EHP442 (PMC5381994; doi:10.1289/EHP442)
Supplement: (2.5 MB) PDF [file EHP442.s001.acco.pdf]

Environ Health Perspect
DOI: 10.1289/EHP442

Note to readers with disabilities: *EHP* strives to ensure that all journal content is accessible to all readers. However, some figures and Supplemental Material published in *EHP* articles may not conform to 508 standards due to the complexity of the information being presented. If you need assistance accessing journal content, please contact ehponline@niehs.nih.gov. Our staff will work with you to assess and meet your accessibility needs within 3 working days.

Supplemental Material

Population-Level Exposure to Particulate Air Pollution during Active Travel: Planning for Low-Exposure, Health-Promoting Cities

Steve Hankey, Greg Lindsey, and Julian D. Marshall

Table of Contents

Summary of land use variables and output of spatial models

Table S1. Summary of land use variables

Table S2. Descriptive statistics of model outputs

"Sweet-spot" neighborhoods by pollutant

Figure S1. Neighborhood-types for particle number concentration. Maps were created in ArcMap; underlying street and land use data are from https://gisdata.mn.gov/.

Figure S2. Neighborhood-types for black carbon concentration. Maps were created in ArcMap; underlying street and land use data are from https://gisdata.mn.gov/.

Figure S3. Neighborhood-types for $PM_{2.5}$ concentration. Maps were created in ArcMap; underlying street and land use data are from https://gisdata.mn.gov/.

Results of spatial models stratified by land use and transportation network variables

Figure S4. Bicycle and pedestrian traffic volumes and particulate concentrations stratified by road type. This plot includes off-street trails unlike Figure 3 in the main text.

Figure S5. Bicycle and pedestrian traffic volumes and particulate concentrations stratified by population density

Figure S6. Bicycle and pedestrian traffic volumes and particulate concentrations stratified by land use mix

Figure S7. Bicycle and pedestrian traffic volumes and particulate concentrations stratified by open space area

Figure S8. Bicycle and pedestrian traffic volumes and particulate concentrations stratified by retail area

Figure S9. Bicycle and pedestrian traffic volumes and particulate concentrations stratified by population density, land use mix, and open space area. Concentrations shown as absolute values instead of normalized values (Figure 4 in the main text)

Figure S10. Bicycle and pedestrian traffic volumes and particulate concentrations stratified by industrial area

Figure S11. Bicycle and pedestrian traffic volumes and particulate concentrations stratified by share of non-white residents

Figure S12. Bicycle and pedestrian traffic volumes and particulate concentrations stratified by household income

Comparison of afternoon rush-hour bicycle and pedestrian traffic with morning rush-hour particulate concentrations

Figure S13. Replication of Figure 1 (main text) using a morning rush-hour (7-9am) concentration surface. Spatial patterns of each factor (left-panel); plots of the transect (right-panel). The plots follow the transect from point A (left) to point B (right). Maps were created in ArcMap; underlying street and land use data are from https://gisdata.mn.gov/.

Figure S14. Replication of Figure 2 (main text) using a morning rush-hour (7-9am) concentration surface. The maps represent the four categories of neighborhood-type outlined in Table 2 of the main text. Maps were created in ArcMap; underlying street and land use data are from https://gisdata.mn.gov/.

Figure S15. Replication of Figure 3 (main text) using a morning rush-hour (7-9am) concentration surface. Trends reflect similar patterns to afternoon rush-hour with more blocks meeting the 15% reduction in BC or PN criteria for the morning surface. Maps were created in ArcMap; underlying street and land use data are from https://gisdata.mn.gov/.

Summary of land use variables and output of spatial models

We explored how the output of our spatial models of bicycle traffic, pedestrian traffic, and particulate air pollution varied by land use variables that may be important for designing health-promoting cities. We stratified our model output by 5 land use variables (two that are commonly cited as effective strategies for increasing "walkability" or "bikeability"; three that were significant in our facility-demand models). Table S1 gives a summary of these variables, how they were assembled, and summary statistics. As shown in Table S1 many locations had a value of "0" for open space and retail area (due to the small buffer size in the facility demand models). We randomized the "0" values for the purpose of plotting trends by quartile. This ensured that quartiles 1 and 2 for those two land use factors generated nearly the same values in the subsequent plots.

Table S1. Summary of land use variables

**Land use variable**	**Unit**	**Spatial scale**	**Reason for stratifying model outputs by land use factor**	**Mean**	**Median**	**P25**	**P75**
Population density	people km^{-2}	Block group	Commonly cited planning goal	2,917	2,622	1,861	3,489
Land use mix	0-1 index	Block group	Commonly cited planning goal	0.44	0.45	0.28	0.58
Open space area	m^{-2}	Network buffer (100 m)	Significant in facility-demand model	3,529	0	0	1,849
Retail area	m^{-2}	Network buffer (100 m)	Significant in facility-demand model	1,326	0	0	670
Industrial area	m^{-2}	Network buffer (1,250 m)	Significant in facility-demand model	140,205	34,904	0	191,163

We calculated summary statistics for our model output. Table S2 shows summary statistics for all 13,604 blocks where spatial estimates were made for each health determinant (active travel; particulate air pollution). All data collected to develop these models were collected in the autumn and during afternoon (4-6pm) rush-hour. As such our results should be interpreted as relevant for that time period and season (see below for a sensitivity analysis that compares the afternoon-based active travel estimates with morning rush-hour particulate concentrations).

Table S2. Descriptive statistics of model outputs[a]

**Parameter**	**Unit**	**Mean**	**Median**	**P5**	**P10**	**P25**	**P75**	**P90**	**P95**
Bicycle traffic	2-hour count	46	27	13	14	16	53	91	130
Pedestrian traffic	2-hour count	55	30	14	16	21	50	91	140
Particle number	pt cm^{-3}	14,057	13,716	12,499	12,626	12,990	14,785	16,081	16,850
Black carbon	µg m^{-3}	0.75	0.72	0.56	0.59	0.64	0.83	0.97	1.06
$PM_{2.5}$	µg m^{-3}	8.5	8.5	8.2	8.2	8.4	8.7	8.9	9.0

[a] All measurements and models are for afternoon rush-hour (4-6pm). Descriptive statistics are based on all city-blocks (n=13,604).

“Sweet-spot” neighborhoods by pollutant

We identified four neighborhood types that result from combinations of the high/low quartiles of active travel and particulate concentrations. Figure 2 in the main text shows an aggregate map for city-blocks that meet inclusion criteria for two of the three pollutants (particle number, black carbon, $PM_{2.5}$). Figures S1-S3 show the same maps but for each pollutant separately.

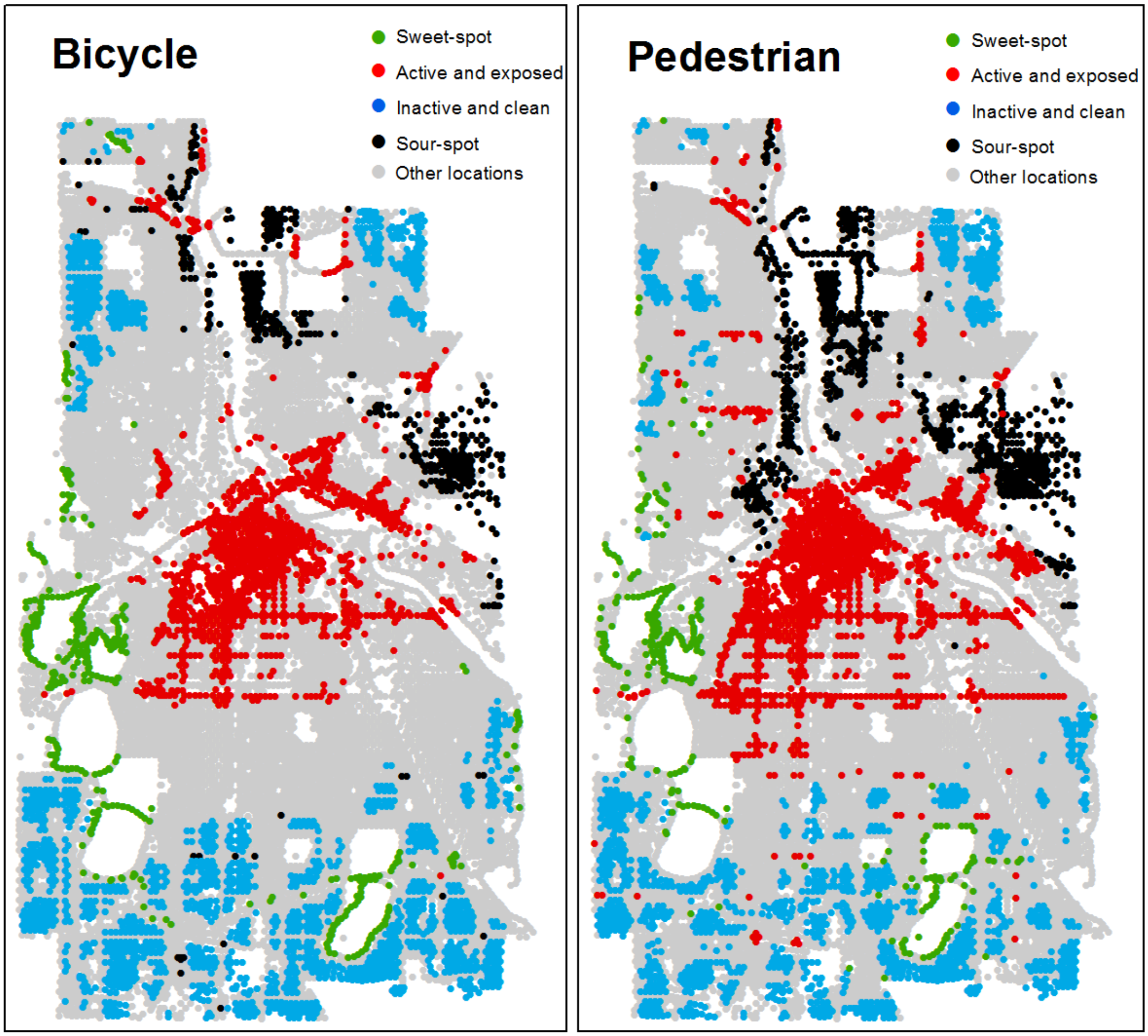

Figure S1. Neighborhood-types for particle number concentration. Maps were created in ArcMap; underlying street and land use data are from https://gisdata.mn.gov/.

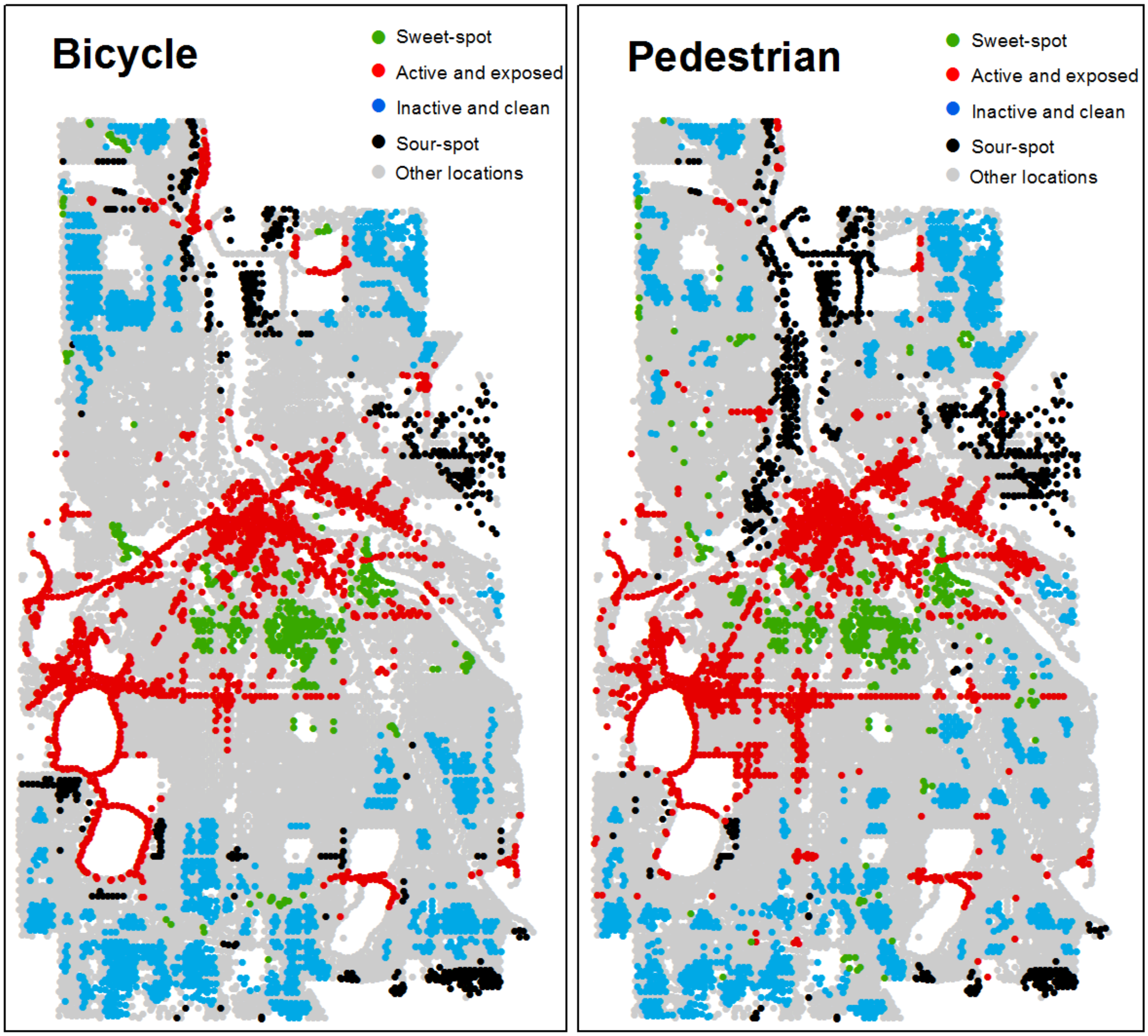

Figure S2. Neighborhood-types for black carbon concentration. Maps were created in ArcMap; underlying street and land use data are from https://gisdata.mn.gov/.

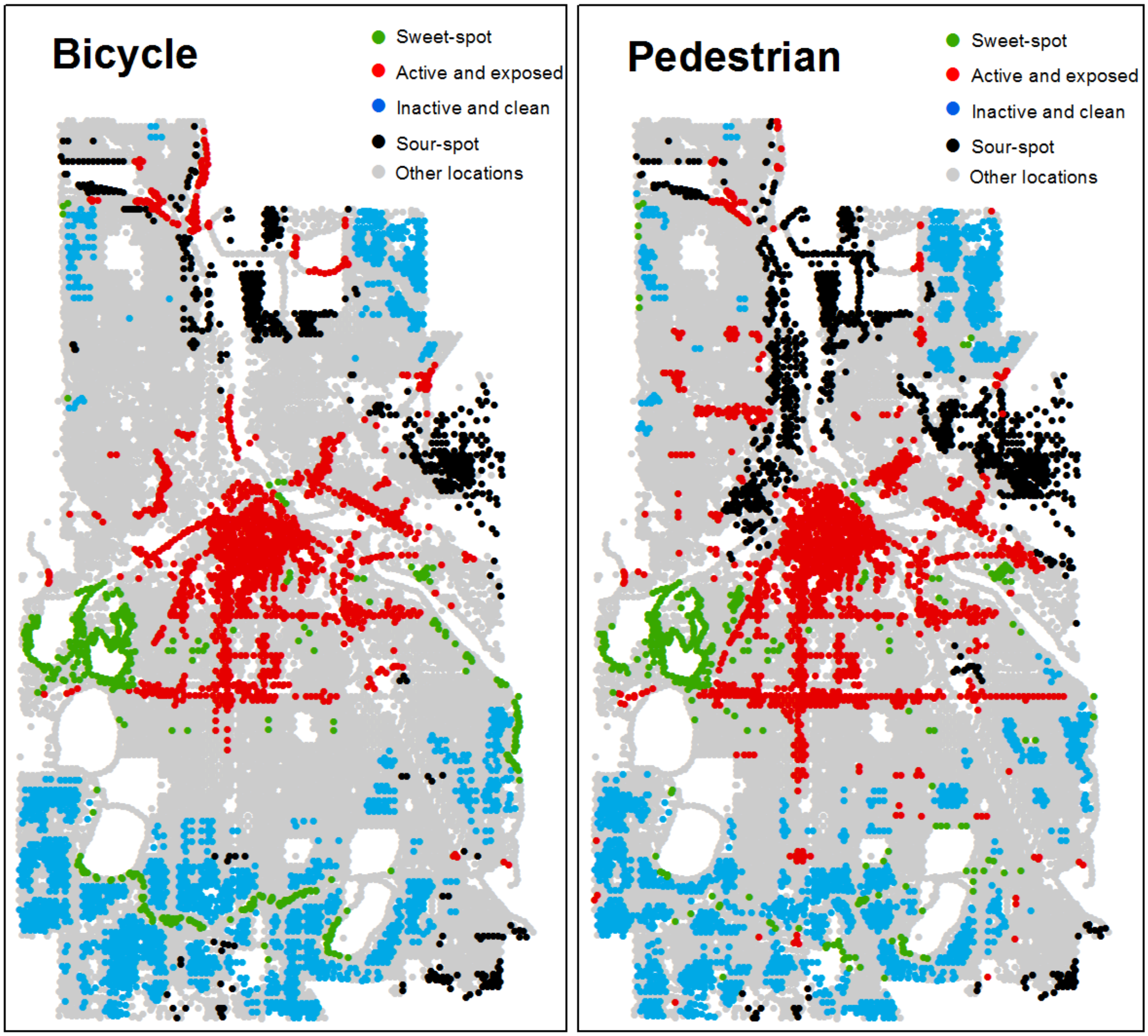

Figure S3. Neighborhood-types for $PM_{2.5}$ concentration. Maps were created in ArcMap; underlying street and land use data are from https://gisdata.mn.gov/.

Results of spatial models stratified by land use and transportation network variables

We stratified our spatial estimates of active travel and particulate air pollution by (1) various land use variables (see Table S1), (2) aspects of the transportation network, and (3) metrics of environmental justice. Figures 3 and 4 in the main text show the core findings for these analyses. However, in the following figures we show each factor separately and include $PM_{2.5}$ ($PM_{2.5}$ demonstrated limited variability and thus was not included in the plots in the main text). Below is a brief summary of each plot:

- Figure S4. A plot similar to Figure 3 of the main text (street functional class) but also including values for off-street trails.
- Figures S5-S8. A separate plot for each factor in Figure 4 of the main text and including $PM_{2.5}$.
- Figure S9. The same as Figure 4 in the main text except air pollution concentrations are shown on an absolute scale.
- Figures S10. Active travel and particulate concentrations by industrial area.
- Figures S11-S12. Active travel and particulate concentrations by median household income and percent of non-white residents.

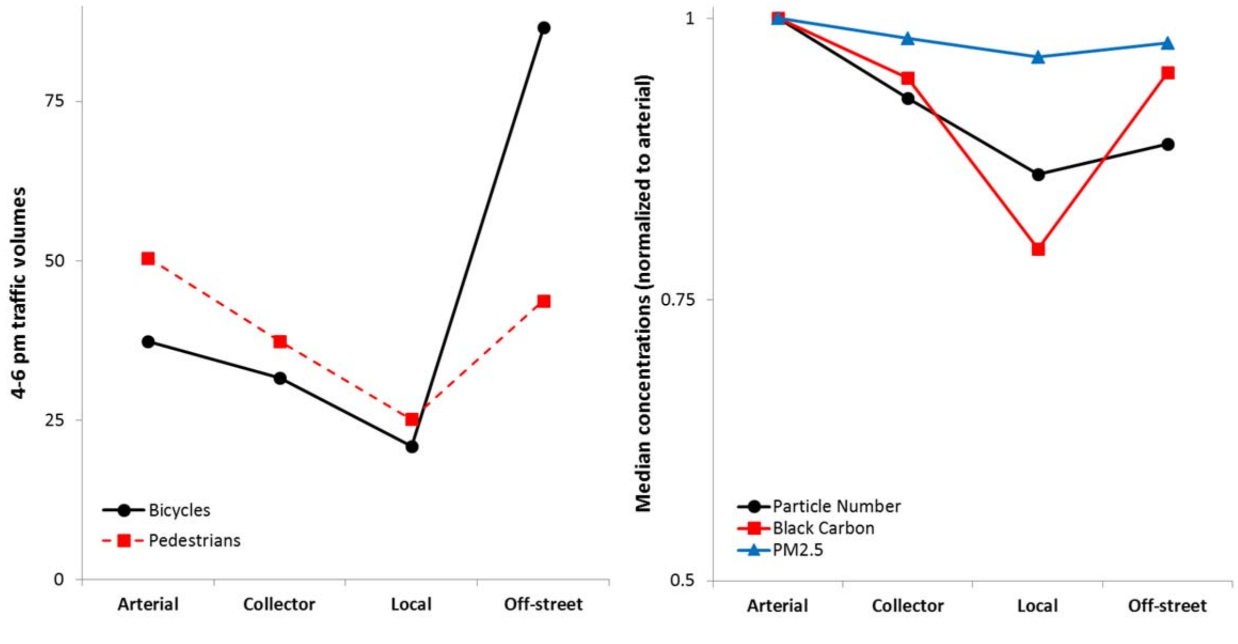

Figure S4. Bicycle and pedestrian traffic volumes and particulate concentrations stratified by road type. This plot includes off-street trails unlike Figure 3 in the main text.

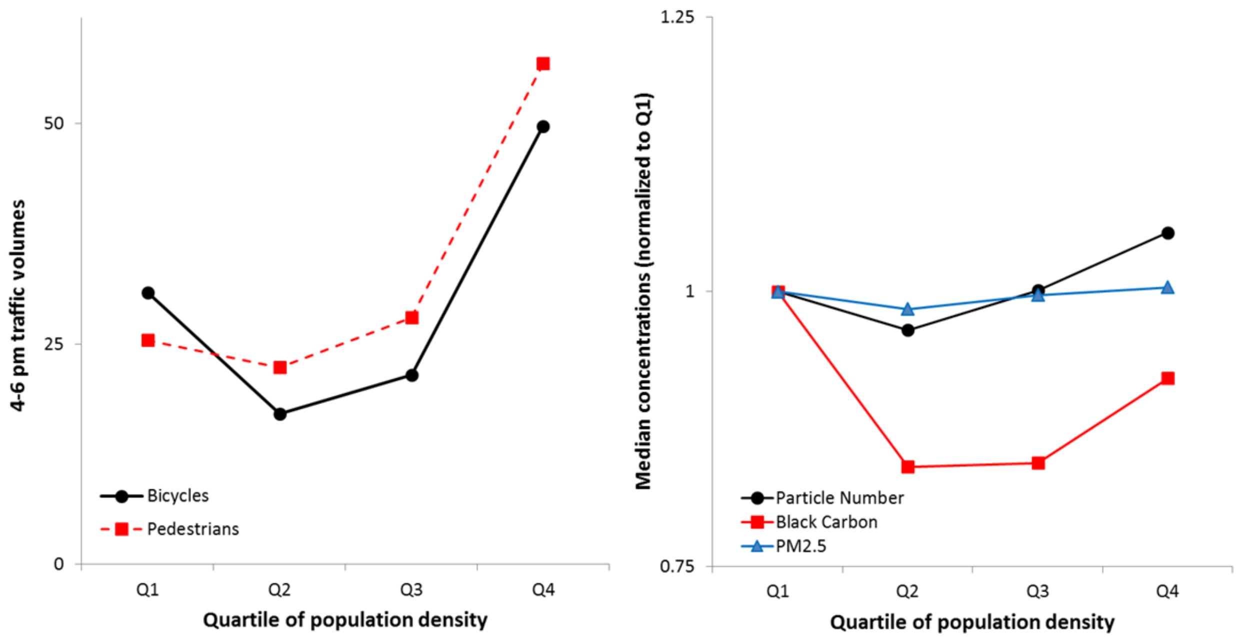

Figure S5. Bicycle and pedestrian traffic volumes and particulate concentrations stratified by population density.

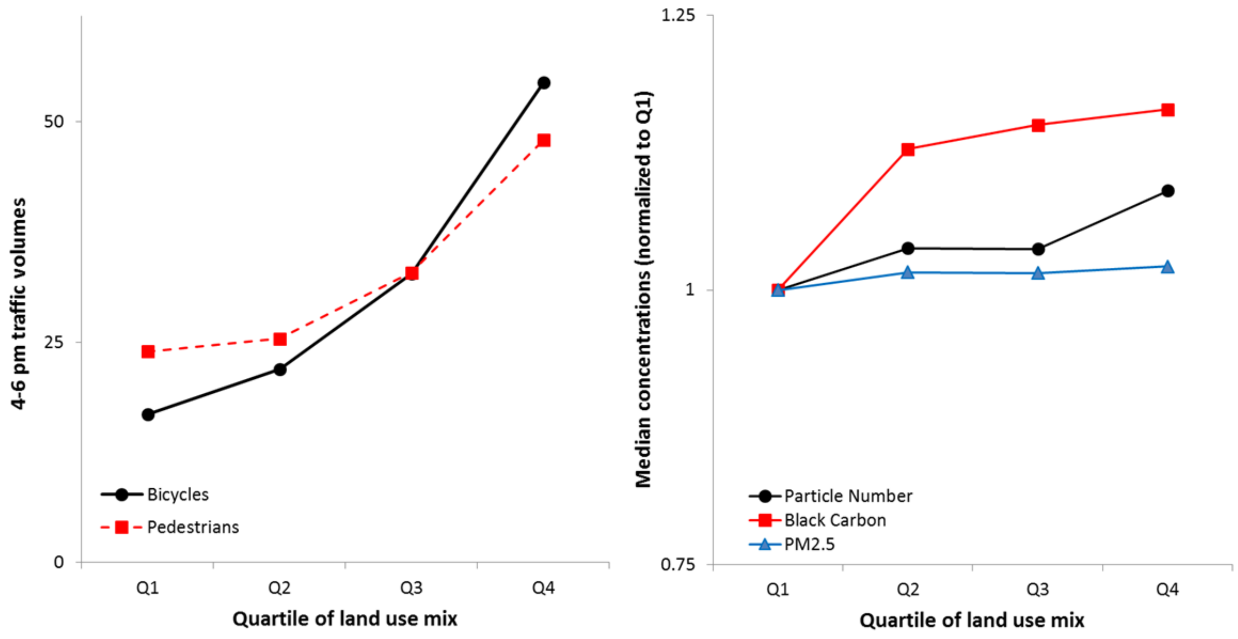

Figure S6. Bicycle and pedestrian traffic volumes and particulate concentrations stratified by land use mix.

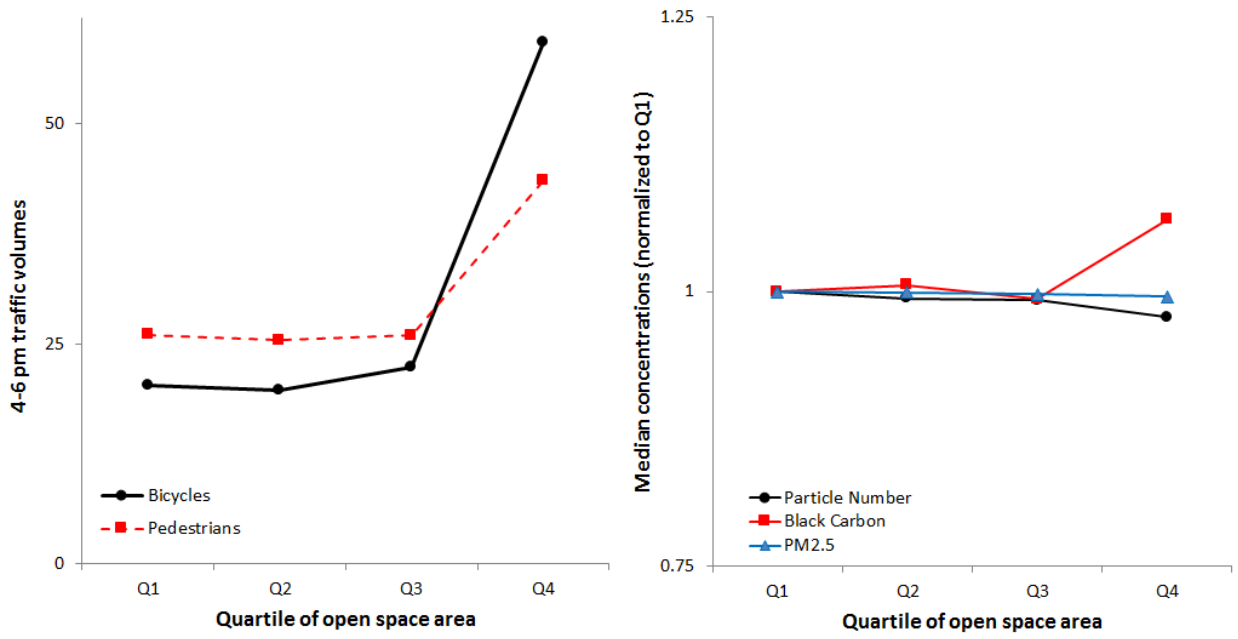

Figure S7. Bicycle and pedestrian traffic volumes and particulate concentrations stratified by open space area.

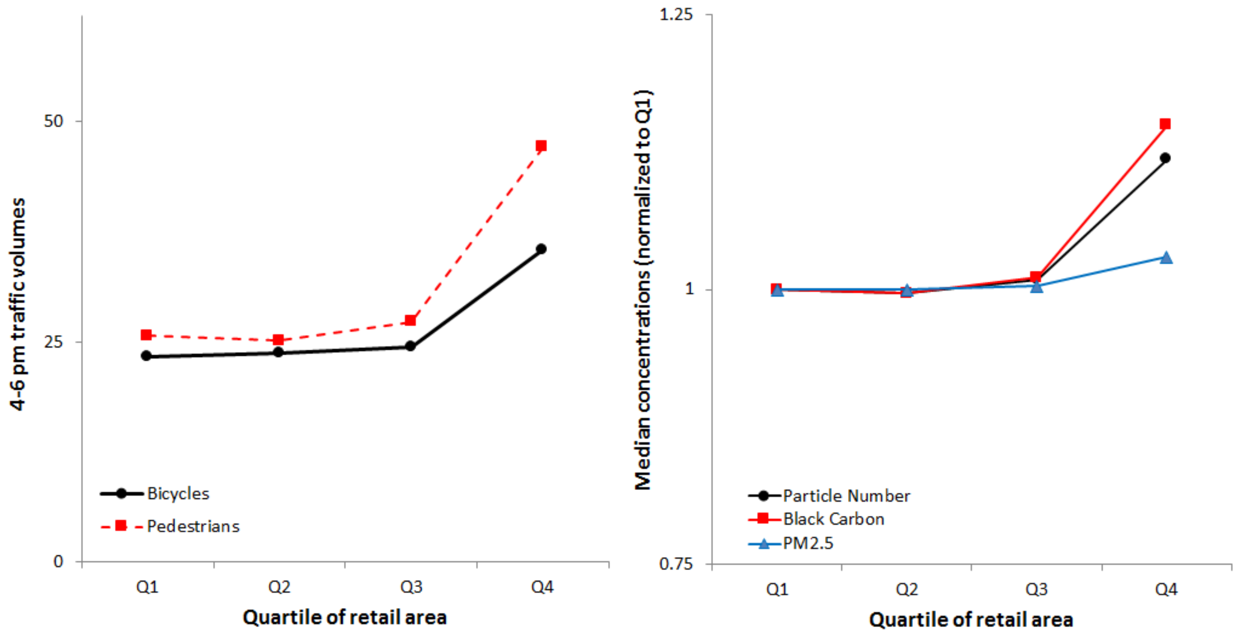

Figure S8. Bicycle and pedestrian traffic volumes and particulate concentrations stratified by retail area.

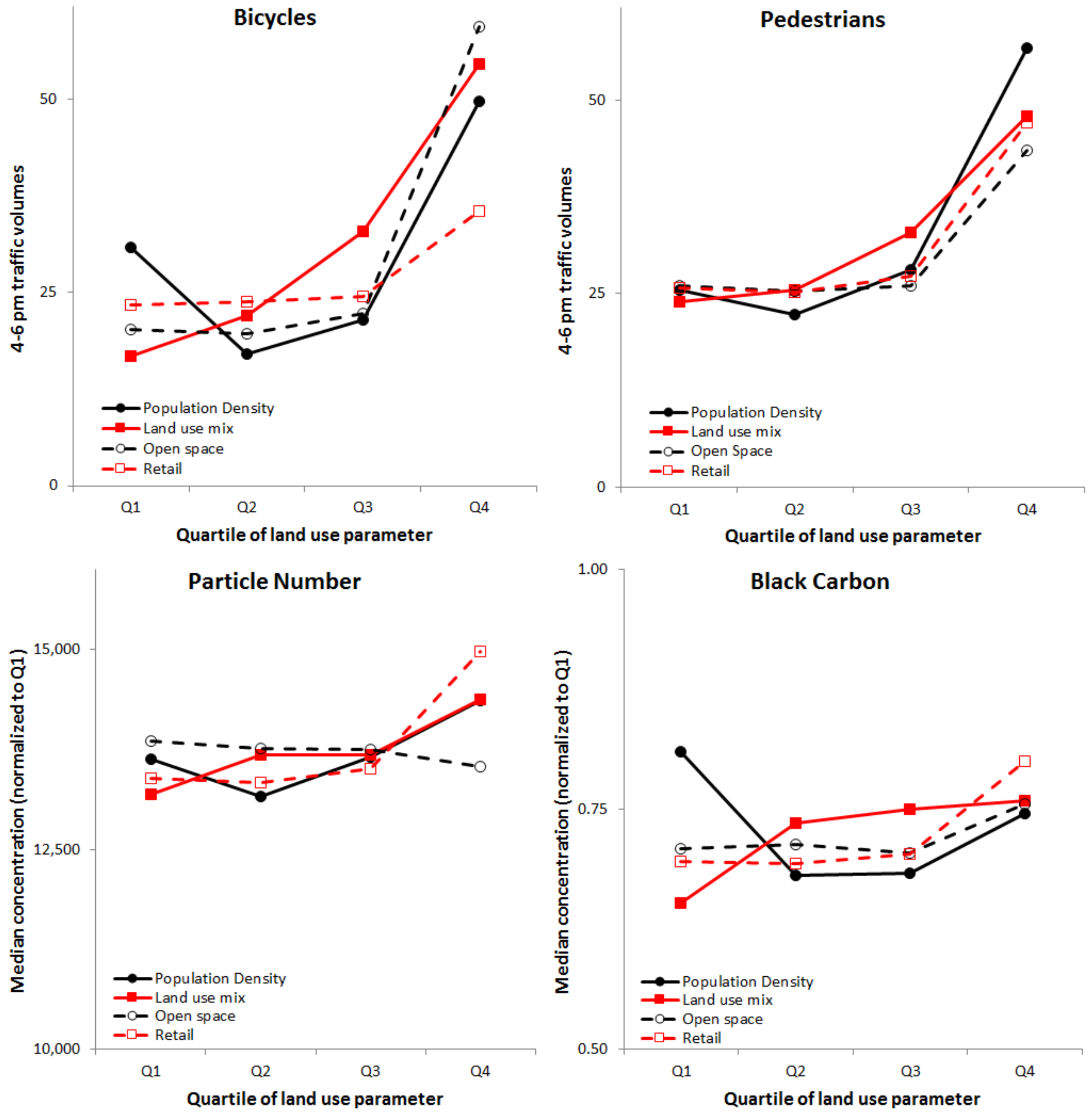

Figure S9. Bicycle and pedestrian traffic volumes and particulate concentrations stratified by population density, land use mix, and open space area. Concentrations shown as absolute values instead of normalized values (Figure 4 in the main text).

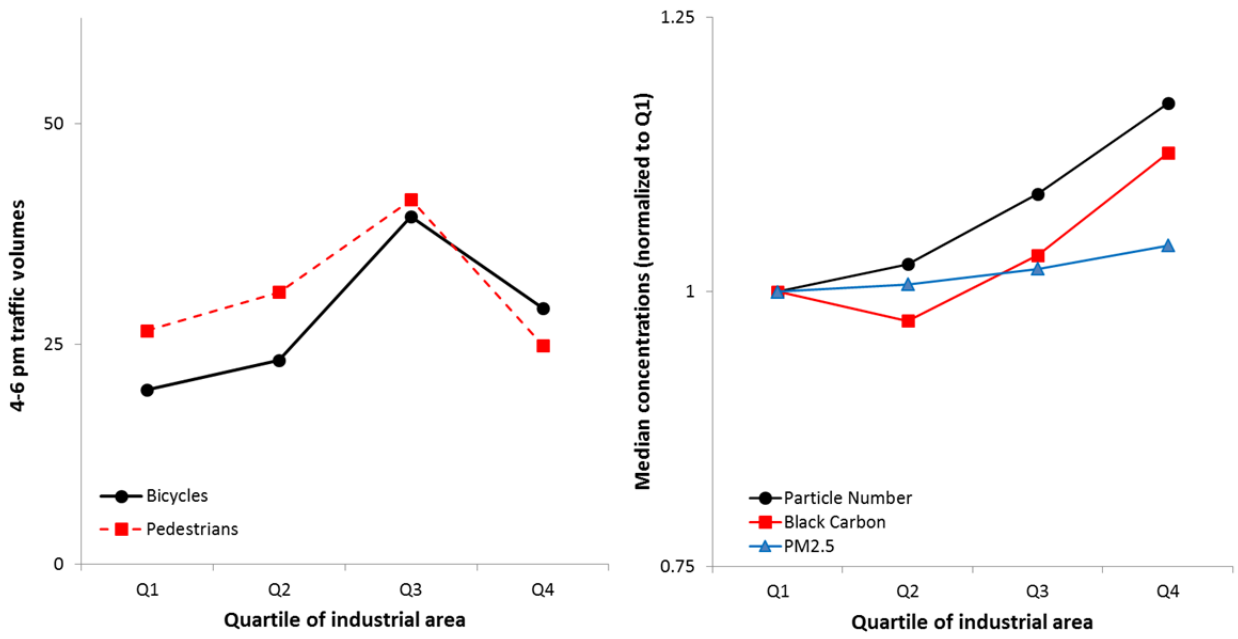

Figure S10. Bicycle and pedestrian traffic volumes and particulate concentrations stratified by industrial area.

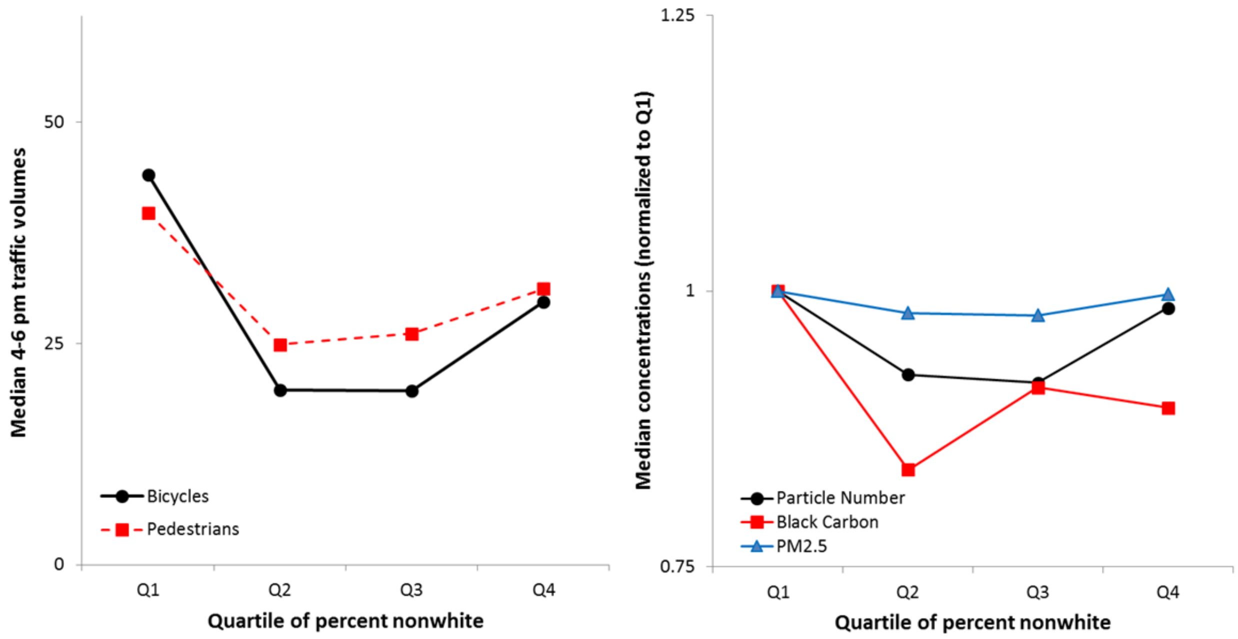

Figure S11. Bicycle and pedestrian traffic volumes and particulate concentrations stratified by share of non-white residents.

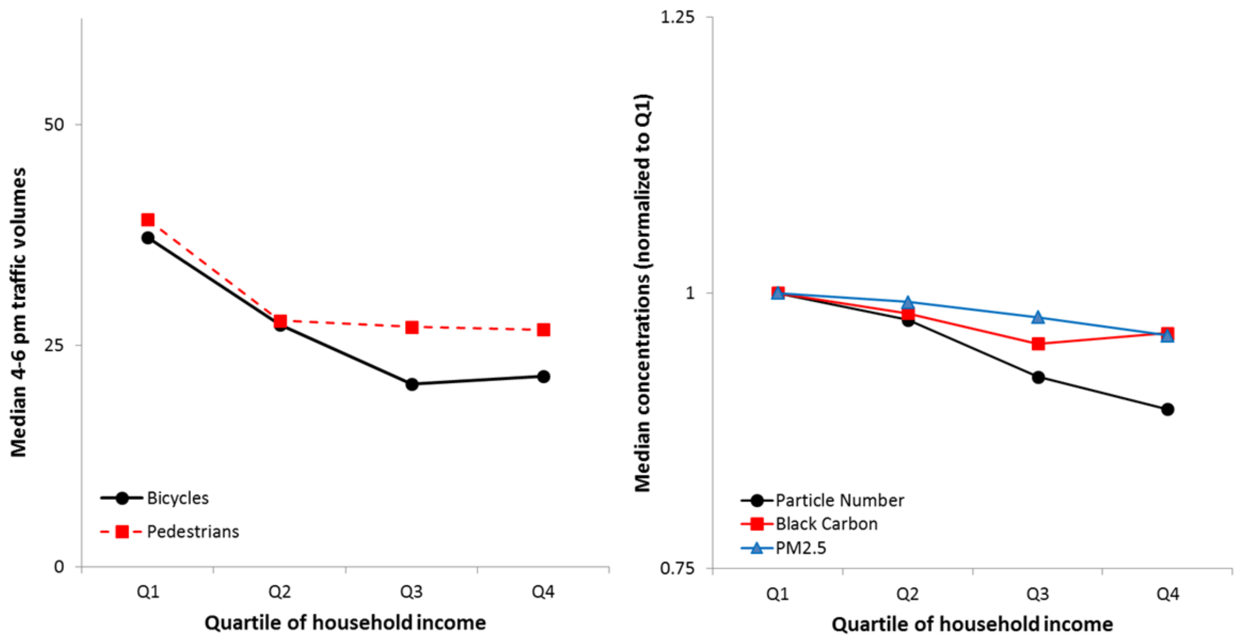

Figure S12. Bicycle and pedestrian traffic volumes and particulate concentrations stratified by household income.

Comparison of afternoon rush-hour bicycle and pedestrian traffic with morning rush-hour particulate concentrations

As a sensitivity analysis we explored using a morning rush-hour (7-9am) particulate concentration surface as an input to our spatial analysis (i.e., comparing spatial patterns of morning concentrations to the afternoon bicycle and pedestrian traffic estimates). To illustrate the findings of this analysis we replicated Figures 1-3 of the main text using the morning particulate surface (see Figure S13-S15). In general, concentrations were higher in the mornings (as compared to the afternoon) and demonstrated more spatial variability. However, the core findings of our base-case reported in the main text (afternoon concentrations) remained unchanged. The greater spatial variability in morning concentrations mostly exacerbated the core findings from the main text indicating that our results may be conservative when comparing to other times of day. A useful direction for future research would be to explore patterns of exposure for different time periods including times of day and seasons.

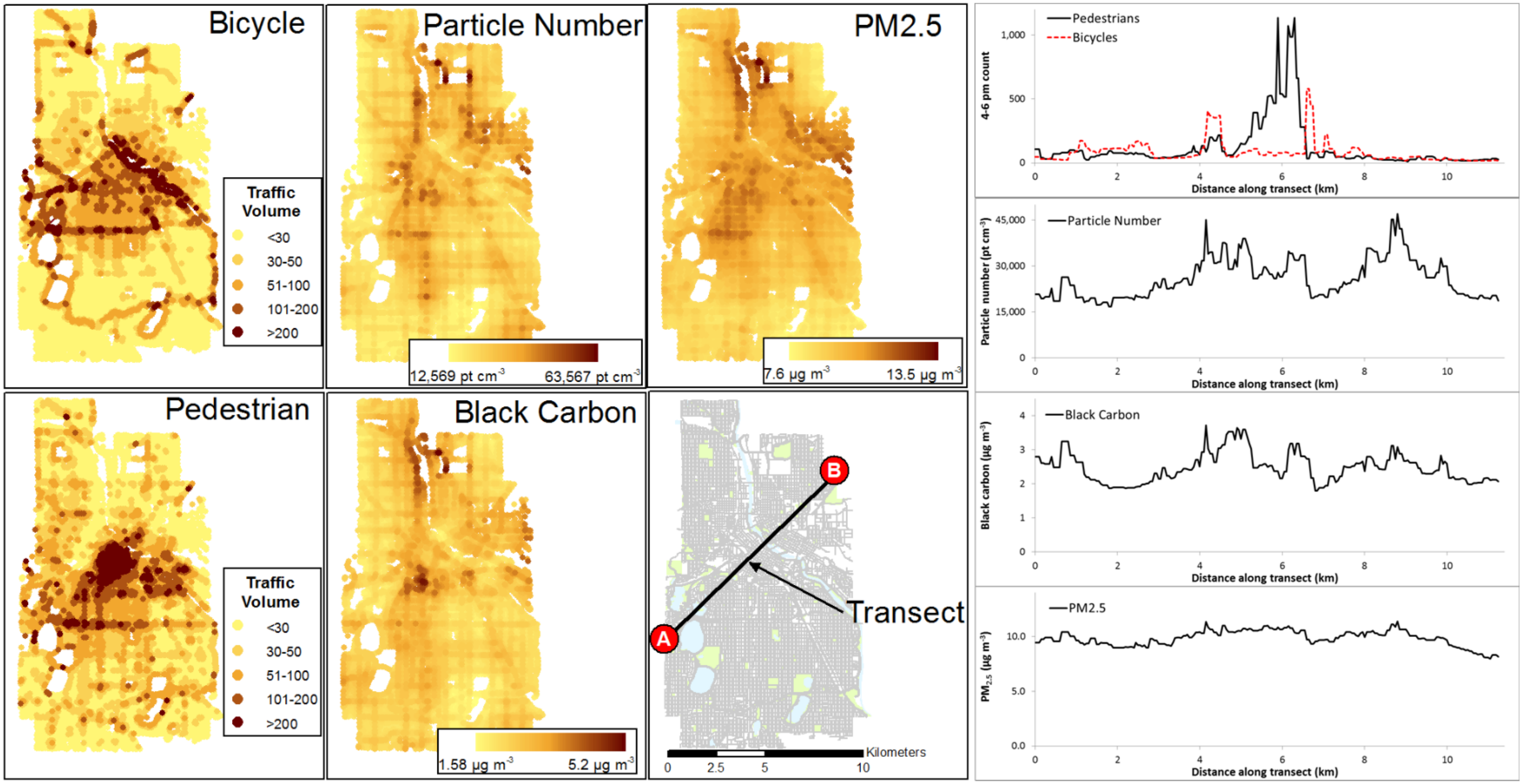

Figure S13. Replication of Figure 1 (main text) using a morning rush-hour (7-9am) concentration surface. Spatial patterns of each factor (left-panel); plots of the transect (right-panel). The plots follow the transect from point A (left) to point B (right). Maps were created in ArcMap; underlying street and land use data are from https://gisdata.mn.gov/.

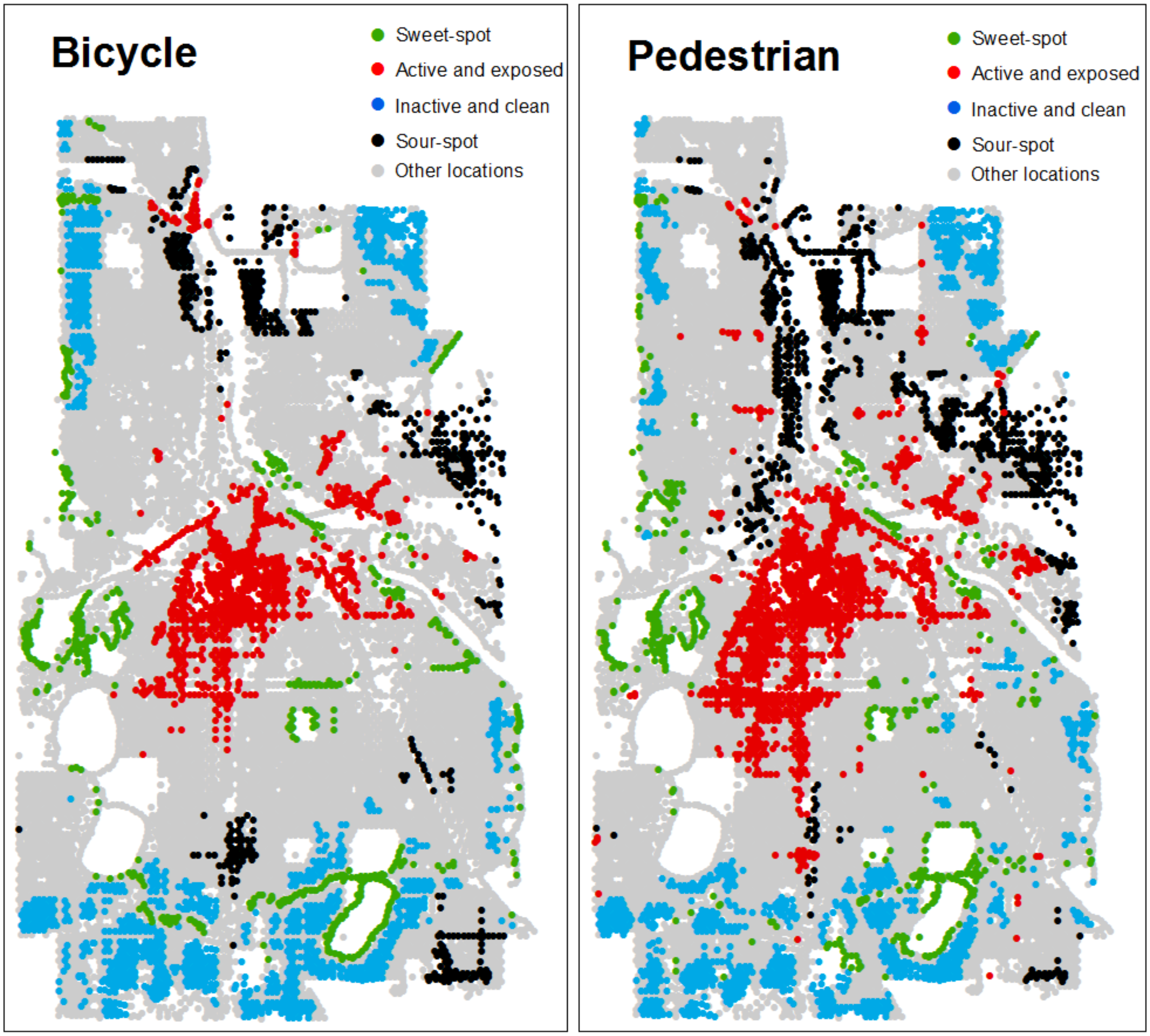

Figure S14. Replication of Figure 2 (main text) using a morning rush-hour (7-9am) concentration surface. The maps represent the four categories of neighborhood-type outlined in Table 2 of the main text. Maps were created in ArcMap; underlying street and land use data are from https://gisdata.mn.gov/.

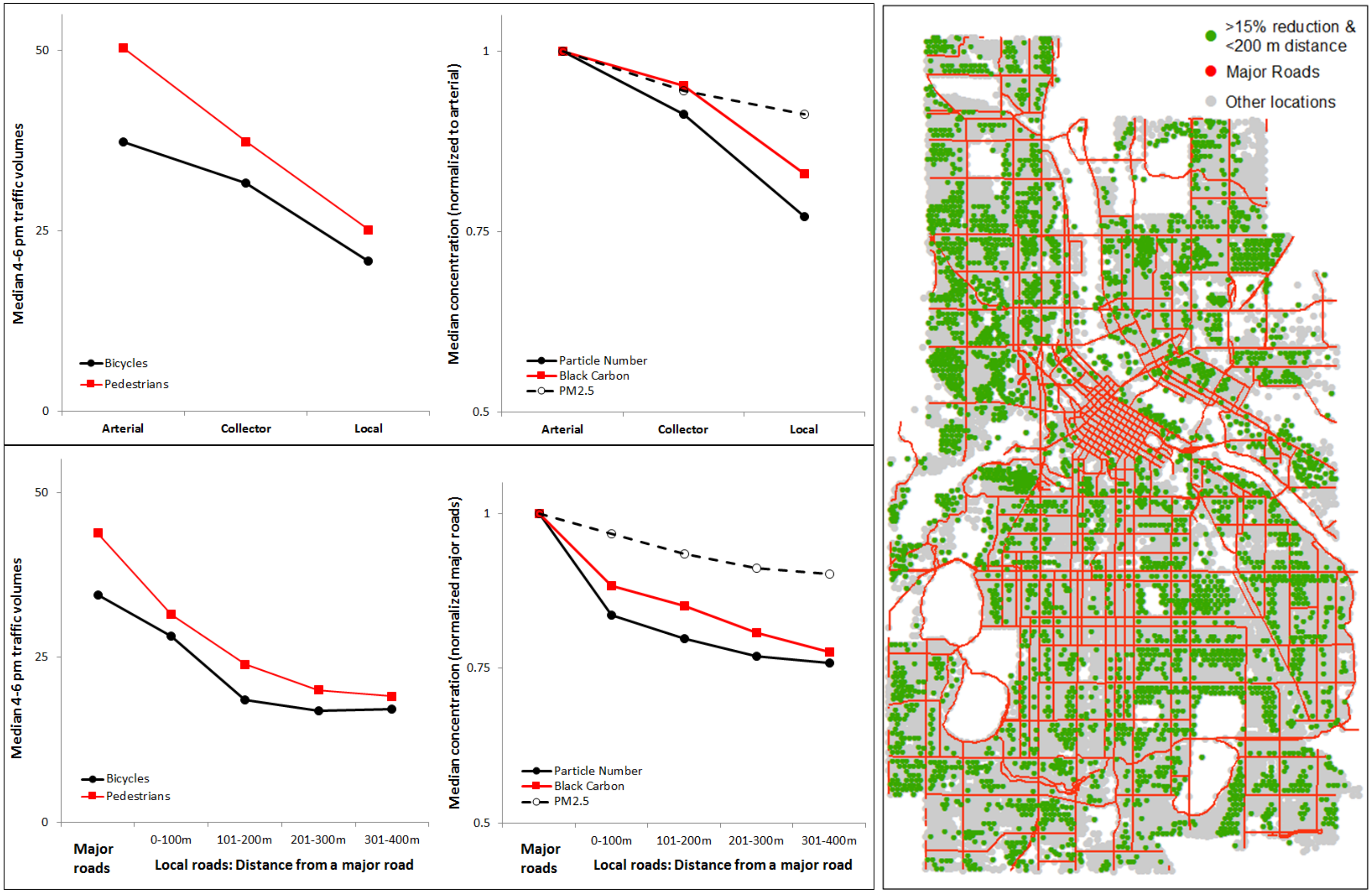

Figure S15. Replication of Figure 3 (main text) using a morning rush-hour (7-9am) concentration surface. Trends reflect similar patterns to afternoon rush-hour with more blocks meeting the 15% reduction in BC or PN criteria for the morning surface. Maps were created in ArcMap; underlying street and land use data are from https://gisdata.mn.gov/.
